# Future work self-salience and perceived professional benefits among nursing interns in China: the indirect roles of clinical belongingness and deep acting in a cross-sectional structural equation modeling study

**DOI:** 10.3389/fmed.2026.1873837

**Published:** 2026-08-28

**Authors:** Ruoyu Du, Yamei Zhang, Qiuyue Cui, Ying Zhong, Man Zhang, Yuan Ge

**Affiliations:** 1 School of Nursing, Guangxi University of Chinese Medicine, Nanning, China; 2 The First Affiliated Hospital of Guangxi University of Chinese Medicine, Nanning, China

**Keywords:** structural equation modeling, clinical belongingness, deep acting, emotional labor, future work self-salience, nursing interns, perceived professional benefits

## Abstract

**Objective:**

This study examined whether future work self-salience was associated with perceived professional benefits among nursing interns and whether clinical belongingness and deep acting were involved as independent and serial mediators in this association.

**Methods:**

A cross-sectional online survey was conducted among 641 nursing interns recruited from six higher education institutions in Guangxi, China (March–April 2026). Measures included future work self-salience (4 items), clinical belongingness (17 items), deep acting (3 items), and perceived professional benefits (29 items). Descriptive statistics, univariate analyses, and correlation analyses were performed. A structural equation model was estimated and a bootstrap procedure with 5,000 resamples tested indirect associations while adjusting for demographic covariates.

**Results:**

All study variables were positively correlated (all *p* < 0.01). The chain mediation model demonstrated good fit (*χ*^2^/df = 2.73, RMSEA = 0.05, GFI = 0.99, AGFI = 0.96, TLI = 0.96, CFI = 0.99, NFI = 0.98). Future work self-salience had a significant direct association with perceived professional benefits (*B* = 1.146, 95% CI: 0.891–1.401). Significant indirect associations were observed through clinical belongingness (*B* = 1.251, 95% CI: 0.963–1.553), through deep acting (*B* = 0.045, 95% CI: 0.005–0.107), and through the sequential pathway of clinical belongingness and deep acting (*B* = 0.039, 95% CI: 0.009–0.084). The total indirect association accounted for 53.81% of the total association.

**Conclusion:**

Future work self-salience was associated with higher perceived professional benefits among nursing interns, both directly and indirectly through clinical belongingness and deep acting. These findings are consistent with the hypothesized serial mediation model, although causal inferences cannot be made due to the cross-sectional design.

## 1 Introduction

Nursing internship is a critical transitional stage during which students move from classroom-based learning to real clinical practice and begin to form more concrete understandings of their future professional roles (1–3). During this period, interns must cope with complex patient needs, interpersonal demands, heavy workloads, and the emotional challenges of clinical care (4–7). How they interpret and derive meaning from these experiences may have important implications for their professional development and retention in nursing (8). Perceived professional benefits (PPB) refers to the positive gains individuals perceive from engaging in their profession, such as personal growth, professional value, self-efficacy, recognition, and a sense of achievement (9). Among nursing students and interns, higher PPB has been associated with stronger professional identity, better adaptation, and more positive career attitudes (10–12). Therefore, identifying factors associated with PPB may help clarify how nursing interns maintain positive professional appraisals during a demanding developmental stage.

Future work self-salience (FWSS) may be one such factor. FWSS refers to the clarity and vividness with which individuals imagine their future work-related selves and career aspirations (13). A salient future work self provides direction, meaning, and motivational focus, helping individuals connect present efforts with desired future roles (14–16). For nursing interns, having a clearer image of the kind of nurse they wish to become may make clinical experiences feel more purposeful and personally relevant, even when those experiences are stressful (17, 18). In this way, FWSS may be associated with stronger perceptions of professional growth, value, and gain (19, 20). Prior studies in career development and vocational psychology suggest that a clearer future-oriented occupational self is linked to greater career adaptability, proactive behavior, goal pursuit, and work-related meaning, all of which may contribute to more positive professional evaluations (21). Accordingly, FWSS may be positively associated with PPB among nursing interns.

A second question concerns how FWSS may be linked to professional appraisals in clinical settings. One plausible pathway is through clinical belongingness. Clinical belongingness refers to the extent to which nursing students or interns feel accepted, included, respected, and supported within the clinical environment (22, 23). It reflects more than simple social comfort; it also signals whether interns feel that they are legitimate participants in the clinical community (24). Interns with a more salient future work self may be more likely to view themselves as emerging members of the nursing profession rather than temporary outsiders. This future-oriented professional image may increase their willingness to seek interaction, build relationships with preceptors and peers, and actively integrate into the clinical environment (14, 15). Through these processes, they may experience stronger belongingness in practice settings. Existing research has shown that goal clarity, career motivation, and proactive engagement are associated with greater relational investment and social adjustment in training contexts, while weak future orientation may reduce engagement and heighten feelings of isolation (25–27). Thus, clinical belongingness may serve as an important mediator linking FWSS to PPB.

Clinical belongingness may also be relevant to how interns regulate emotions in patient care. In emotionally demanding contexts such as nursing, emotional labor is often required to display appropriate professional emotions (28). However, emotional labor is multidimensional, and its dimensions may have different implications. In particular, deep acting involves attempting to modify internal feelings so that they align with professional display expectations, whereas surface acting involves merely faking outward emotional expressions without changing inner feelings (29). Compared with surface acting, deep acting is often considered a more internalized and potentially adaptive emotion regulation strategy (30–32). A stronger sense of belongingness may facilitate such internalization. When interns feel included, respected, and psychologically safe within the clinical team, they may be more likely to identify with professional norms and values and to regulate emotions in ways that genuinely support caring interactions (33–36). By contrast, when interns feel excluded or marginalized, emotional expression may be more defensive, effortful, or externally imposed. Therefore, clinical belongingness may be positively associated with deep acting.

Deep acting, in turn, may be associated with higher PPB. Unlike emotional labor treated as a unitary construct, deep acting may carry more positive professional implications because it involves aligning internal emotional experience with professional caring values (37, 38). This process may promote greater authenticity in patient interactions, reduce emotional dissonance, and strengthen a sense of competence and role congruence (31, 39). For nursing interns, engaging in deep acting may help transform emotionally demanding encounters into opportunities for empathy, learning, and professional growth. Previous studies have suggested that deep acting is more likely than surface acting to be associated with adaptive work outcomes, including better interpersonal functioning, stronger professional meaning, and more favorable psychological adjustment (40–42). Therefore, deep acting may represent an explanatory emotional-regulation pathway through which FWSS and clinical belongingness are associated with PPB.

Taken together, these considerations suggest not only independent mediating roles of clinical belongingness and deep acting, but also a possible sequential pathway linking them. Interns with a more salient future work self may be more likely to engage with the clinical environment and experience stronger belongingness; this stronger belongingness may then facilitate internalization of professional emotional display rules and a greater tendency toward deep acting, which in turn may be associated with higher PPB. Although alternative ordering among mediators is possible, we proposed clinical belongingness as preceding deep acting based on the assumption that a stronger sense of inclusion in the clinical environment may facilitate internalization of professional display rules and, in turn, greater use of deep acting. Nevertheless, given the cross-sectional nature of the data, this ordering should be viewed as theoretically informed rather than causally established.

Although previous studies have examined professional identity, clinical learning environment, emotional labor, and perceived professional benefits among nursing students or nurses, less is known about how nursing interns' future-oriented professional self-concept is associated with their current perception of professional benefits. In particular, the potential roles of clinical belongingness and deep acting have not been sufficiently examined within one integrated model. Addressing this gap may help clarify how future work self-salience is linked to positive professional perceptions during the transition from nursing education to clinical practice.

Therefore, this study aimed to examine the associations among future work self-salience, clinical belongingness, deep acting, and perceived professional benefits among nursing interns in Guangxi, China. Specifically, we tested whether clinical belongingness and deep acting were indirectly involved in the association between future work self-salience and perceived professional benefits, both separately and sequentially. Given the cross-sectional design, the proposed model was considered theoretically driven and associational rather than causal.

## 2 Materials and methods

### 2.1 Study design and setting

An institution-based cross-sectional survey was conducted between March and April 2026 among nursing interns from six higher education institutions in Guangxi, China. The study was reported in accordance with the STROBE guidelines for cross-sectional studies. Guangxi was selected as the study setting because it is an important region for nursing education and clinical internship training in southwest China. The six institutions were selected based on established nursing internship programs, accessibility to the research team, and institutional willingness to assist with recruitment.

### 2.2 Participants

Convenience sampling was used. Eligible participants were full-time nursing students enrolled in a recognized nursing program, had completed at least 3 months of clinical internship, and provided informed consent. Students who had been on continuous leave for more than 4 weeks during the survey period were excluded. Because convenience sampling was used, the sample was not designed to be statistically representative of all nursing interns in Guangxi or China. Therefore, the generalizability of the findings should be interpreted cautiously. A total of 686 questionnaires were collected. After excluding 45 invalid questionnaires because of patterned responses or implausibly short completion times, 641 valid questionnaires were retained, yielding an effective response rate of 93.44%.

### 2.3 Procedure and data quality control

Data were collected using an online questionnaire platform. Before data collection, the research team contacted nursing program administrators or internship coordinators at the participating institutions and explained the study purpose and eligibility criteria. Eligible nursing interns received an invitation containing a questionnaire link or QR code. The first page of the questionnaire provided information about the study purpose, voluntary participation, anonymity, confidentiality, and the right to withdraw at any time. Participants could proceed to the survey only after indicating informed consent.

To improve data quality, all items were set as mandatory to avoid missing data, and each internet protocol address was allowed to submit the questionnaire only once. The questionnaire platform automatically recorded completion time. Responses were excluded if they showed obvious patterned responses, such as selecting the same option across nearly all scale items, or if the completion time shorter than 180 s. The specific screening criteria were applied before formal statistical analysis.

### 2.4 Measures

#### 2.4.1 Demographic information

Demographic variables included age, gender, residence, only-child status, student leadership experience, highest educational level, preference for nursing, Average monthly household income, internship duration, and employment outlook.

#### 2.4.2 Future work self-salience

Future self-salience was assessed using the Chinese version of *the Future Work Self Scale* originally developed by Strauss (13) and subsequently revised and validated in the Chinese context by Guan (43). Future self-salience was assessed with a 4-item single-dimension scale. Items were rated on a 5-point Likert scale (1 = strongly disagree to 5 = strongly agree); higher scores indicate greater clarity. Internal consistency in this study was Cronbach's *α* = .909.

#### 2.4.3 Clinical belongingness

Clinical belongingness was measured using the *Nursing Student Belongingness Scale* originally developed by Patel (44). The Chinese version used in this study was translated, culturally adapted, and revised by Wang (45). Clinical belongingness was measured using a 17-item scale with three domains reflecting belongingness with classmates, clinical nurses, and teachers. Items were rated on a 5-point Likert scale, with higher scores indicating stronger belongingness. Cronbach's *α* in this study was.888.

#### 2.4.4 Perceived professional benefits

Perceived professional benefits was assessed using the Chinese version of the scale originally developed by Hu Jing in 2013 (9). The scale comprises 29 items covering five dimensions: positive professional perception, positive nurse–patient relationships, recognition from relatives and friends, sense of team belongingness, and personal growth. Items were rated on a 5-point Likert scale (1 = strongly disagree to 5 = strongly agree), with higher scores indicating higher perceived professional benefits. Cronbach's *α* in this study was.863.

#### 2.4.5 Deep acting

Deep acting was assessed using the Chinese version of the deep acting subscale from the *emotional labor scale* originally developed by Grandey (46). This Chinese version was translated and culturally adapted by Luo et al. (47), and validated among Chinese nurse populations (48). Deep acting subscale, which has been validated in Chinese nursing populations. Deep acting was measured using the 3-item deep acting subscale of a nursing emotional labor scale. Items were rated on a 6-point scale from 0 (strongly disagree) to 5 (strongly agree), with higher scores indicating more frequent deep acting. Cronbach's *α* for the deep acting subscale in this study was.962.

### 2.5 Statistical analysis

Statistical analyses were conducted using SPSS version 27.0 and AMOS version 26.0. Descriptive statistics were used to summarize participant characteristics and study variables. The reliability of each scale was evaluated using Cronbach's *α*. Common method bias was examined using Harman's single-factor test.

Independent-samples *t*-tests and one-way analysis of variance were used as univariate descriptive comparisons to explore whether perceived professional benefits differed across demographic subgroups. These analyses were not used as the sole basis for covariate selection. Instead, covariates were selected based on previous literature, theoretical relevance, and their potential associations with professional development outcomes among nursing interns.

Residence, preference for nursing, academic ranking, Average monthly household income, employment outlook, and highest educational level were included as covariates in the adjusted model because previous studies (19, 37) suggest that highest educational level, socioeconomic conditions, academic performance, career preference, and perceived employment outlook are associated with nursing students' professional identity, professional attitudes, or perceived professional benefits. Therefore, these variables were controlled for in the adjusted models.

A structural equation model was estimated to examine the hypothesized associations among future work self-salience, clinical belongingness, deep acting, and perceived professional benefits. Model fit was evaluated using multiple fit indices, including *χ*^2^/df, CFI, TLI, RMSEA. Indirect associations were tested using a bootstrap procedure with 5,000 resamples. An indirect association was considered statistically significant when the 95% bias-corrected confidence interval did not include zero.

Because the data were cross-sectional, the SEM results were interpreted as associations within a theoretically specified model rather than evidence of causal or temporal relationships.

## 3 Results

### 3.1 Common method bias

Common method bias was assessed using Harman's single-factor test across all questionnaire items. The unrotated exploratory factor analysis yielded 10 factors with eigenvalues greater than 1. The first factor accounted for 24.71% of the total variance, below the conventional 40% threshold, suggesting that common method bias was unlikely to substantively affect the results.

### 3.2 Descriptive statistics and univariate analyses

Among the 641 participants, the mean age was 21.99 ± 1.14 years (range: 18–28), 86.0% were female, 55.2% resided in urban areas, and 66.3% held a bachelor's degree. The mean scores were: FWSS 14.15 ± 3.26, clinical belongingness 61.25 ± 10.27, deep acting 9.82 ± 2.98, and PPB 102.20 ± 14.13, all at moderate levels. Significant differences in scale scores were observed across residence, Average monthly household income, academic ranking, preference for nursing, and employment outlook (all *p* < 0.05). gender and internship duration showed no significant differences across any scale (all *p* > 0.05). Detailed results are presented in Table 1. These analyses were exploratory and descriptive.

**Table 1:** Comparison of general demographic characteristics and scale scores (mean ± SD).

Variable	*n* (%)	Future work self-salience	Clinical belongingness	Deep acting	Perceived professional benefits
Gender					
Male	90 (14.0%)	14.26 ± 3.16	61.24 ± 11.23	9.46 ± 2.94	101.33 ± 14.89
Female	551 (86.0%)	14.13 ± 3.28	61.25 ± 10.12	9.87 ± 2.99	102.34 ± 14.01
*t*-value		0.336	−0.005	−1.236	−0.625
*p*-value		0.737	0.996	0.217	0.532
Residence					
Urban	354 (55.2%)	14.58 ± 3.11	62.59 ± 10.34	10.28 ± 2.92	103.92 ± 14.05
Rural	287 (44.8%)	13.62 ± 3.37	59.60 ± 9.96	9.24 ± 2.97	100.07 ± 13.95
*t*-value		3.752	3.722	4.460	3.456
*p*-value		<0.001	<0.001	<0.001	0.001
Only child					
Yes	171 (26.7%)	14.43 ± 3.39	61.43 ± 11.20	10.30 ± 3.05	103.38 ± 16.18
No	470 (73.3%)	14.05 ± 3.21	61.18 ± 9.92	9.64 ± 2.95	101.77 ± 13.29
*t*-value		1.305	0.272	2.508	1.169
*p*-value		0.192	0.786	0.012	0.243
Student leader					
Yes	254 (39.6%)	14.60 ± 3.30	61.39 ± 11.09	10.39 ± 2.98	103.14 ± 15.21
No	387 (60.4%)	13.85 ± 3.20	61.16 ± 9.71	9.44 ± 2.94	101.58 ± 13.35
*t*-value		2.872	0.280	3.961	1.368
*p*-value		0.004	0.780	<0.001	0.172
Average monthly household income					
<3,000	74 (11.5%)	12.77 ± 3.51	57.81 ± 9.51	9.22 ± 3.28	100.19 ± 11.17
3,000–5,000	228 (35.6%)	14.43 ± 3.40	62.07 ± 10.92	10.49 ± 2.71	103.81 ± 14.40
5,001–8,000	198 (30.9%)	14.02 ± 2.94	60.44 ± 8.33	9.25 ± 2.82	100.45 ± 12.44
>8,000	141 (22.0%)	14.60 ± 3.16	62.87 ± 11.54	9.84 ± 3.27	103.09 ± 16.78
*F*-value		6.134	4.919	7.405	2.700
*p*-value		<0.001	0.002	<0.001	0.045
Internship duration					
≤6months	327 (51.0%)	14.19 ± 3.28	61.35 ± 10.08	9.80 ± 2.99	101.93 ± 13.51
>6months	314 (49.0%)	14.11 ± 3.25	61.14 ± 10.48	9.84 ± 2.98	102.47 ± 14.75
*t*-value		0.303	0.264	−0.180	−0.488
*p*-value		0.762	0.792	0.857	0.626
Highest educational level					
Associate degree	156 (24.3%)	13.78 ± 3.68	58.49 ± 10.27	9.25 ± 3.01	98.18 ± 15.63
Bachelor's degree	425 (66.3%)	14.31 ± 3.16	61.94 ± 9.79	10.02 ± 3.00	103.53 ± 13.74
Master's degree	60 (9.4%)	13.93 ± 2.74	63.50 ± 12.21	9.85 ± 2.63	103.20 ± 10.50
*F*-value		1.658	8.209	3.823	8.548
*p*-value		0.191	<0.001	0.022	<0.001
Academic ranking					
Top 1/3	233 (36.3%)	14.61 ± 3.15	62.52 ± 10.89	10.42 ± 2.98	104.10 ± 14.51
Middle 1/3	350 (54.6%)	13.93 ± 3.24	60.70 ± 9.91	9.55 ± 2.98	101.52 ± 13.71
Bottom 1/3	58 (9.0%)	13.57 ± 3.64	59.48 ± 9.43	8.98 ± 2.63	98.66 ± 14.23
*F*-value		4.078	3.149	8.718	4.386
*p*-value		0.017	0.044	<0.001	0.013
Preference for nursing					
Yes	258 (40.2%)	15.08 ± 2.93	63.89 ± 9.50	11.22 ± 2.63	107.21 ± 11.66
No	383 (59.8%)	13.52 ± 3.33	59.47 ± 10.40	8.87 ± 2.83	98.82 ± 14.64
*t*-value		6.263	5.456	10.787	8.054
*p*-value		<0.001	<0.001	<0.001	<0.001
Employment prospect					
Confident to find job	177 (27.6%)	15.49 ± 3.17	63.70 ± 10.99	10.77 ± 3.15	106.16 ± 15.36
Job secured	161 (25.1%)	14.01 ± 3.22	59.61 ± 11.07	9.32 ± 3.06	99.36 ± 15.19
Worried about finding job	228 (35.6%)	13.61 ± 2.84	60.82 ± 8.83	9.55 ± 2.53	101.93 ± 11.25
Unwilling to work in nursing	75 (11.7%)	12.91 ± 3.76	60.28 ± 9.94	9.43 ± 3.23	99.75 ± 14.72
*F*-value		16.972	5.175	8.915	7.825
*p*-value		<0.001	0.002	<0.001	<0.001

### 3.3 Correlation analysis

After adjusting for demographic covariates (residence, highest educational level, preference for nursing, academic ranking, Average monthly household income, and employment outlook), partial correlations indicated that future work self-salience was positively correlated with clinical belongingness (*r* = .51), deep acting (*r* = .20), and perceived professional benefits (*r* = .57). Clinical belongingness was positively correlated with deep acting (*r* = .24) and perceived professional benefits (*r* = .72), and deep acting was positively correlated with perceived professional benefits (*r* = .28). All correlations were statistically significant (*p* < 0.01; Table 2).

**Table 2:** Correlation matrix among future work self-salience, clinical belongingness, deep acting, and perceived professional benefits in nursing interns (r).

Variable	Future work self-salience	Clinical belongingness	Deep acting	Perceived professional benefits
Future work self-salience	1			
Clinical Belongingness	.51*	1		
Deep Acting	.20*	.24*	1	
Perceived Professional Benefits	.57*	.72*	.28*	1

* Results were controlled for residence, preference for nursing, academic ranking, Average monthly household income, employment prospect, and highest education level. *p* < 0.01.

### 3.4 Model fit and mediation analysis

The structural equation model demonstrated good fit: *χ*^2^/df = 2.73, RMSEA = 0.05, GFI = 0.99, AGFI = 0.96, TLI = 0.96, CFI = 0.99, NFI = 0.98 (Table 3).

**Table 3:** Fit indices of the chain mediation structural equation model.

Index	*Χ*^2^/df	RMSEA	GFI	AGFI	TLI	CFI	NFI
Model fit	2.73	0.05	0.99	0.96	0.96	0.99	0.98
Criterion	≤3.00	<0.08	>0.90	>0.90	>0.90	>0.90	>0.90

RMSEA, root mean square error of approximation; GFI, goodness-of-fit index; AGFI, adjusted goodness-of-fit index; TLI, Tucker–Lewis index; CFI, comparative fit index; NFI, normed fit index.

Bootstrap analysis (Table 4) revealed that the total effect of FWSS on PPB was 2.481, of which the direct effect was 1.146 (46.19%) and the total indirect effect was 1.335 (53.81%). Three specific indirect pathways were identified:

Path 1 (FWSS → Clinical belongingness → PPB): B = 1.251, 95% CI: 0.963–1.553, accounting for 50.43% of the total effect.
Path 2 (FWSS → Deep Acting → PPB): B = 0.045, 95% CI: 0.005–0.107, accounting for 1.80% of the total effect.
Path 3 (FWSS → Clinical belongingness → Deep Acting → PPB): B = 0.039, 95% CI: 0.009–0.084, accounting for 1.58% of the total effect.

**Table 4:** Bootstrap analysis of the mediating effects of clinical belongingness and deep acting between future work self-salience and perceived professional benefits.

Path	Effect (95% CI)	SE	Proportion (%)
FWSS → Clinical Belongingness → PPB	1.251 (0.963–1.553)	0.153	50.43
FWSS → Deep Acting → PPB	0.045 (0.005–0.107)	0.027	1.80
FWSS → Clinical Belongingness → Deep Acting → PPB	0.039 (0.009–0.084)	0.019	1.58
Total indirect effect	1.335 (1.032–1.646)	0.159	53.81
Direct effect	1.146 (0.891–1.401)	0.130	46.19
Total effect	2.481	–	100.00

All analyses controlled for residence, highest education level, preference for nursing, academic ranking, Average monthly household income, and employment prospect. Bootstrap resampling = 5,000 times. 95% CI not containing 0 indicates significance.

**Figure 1:**
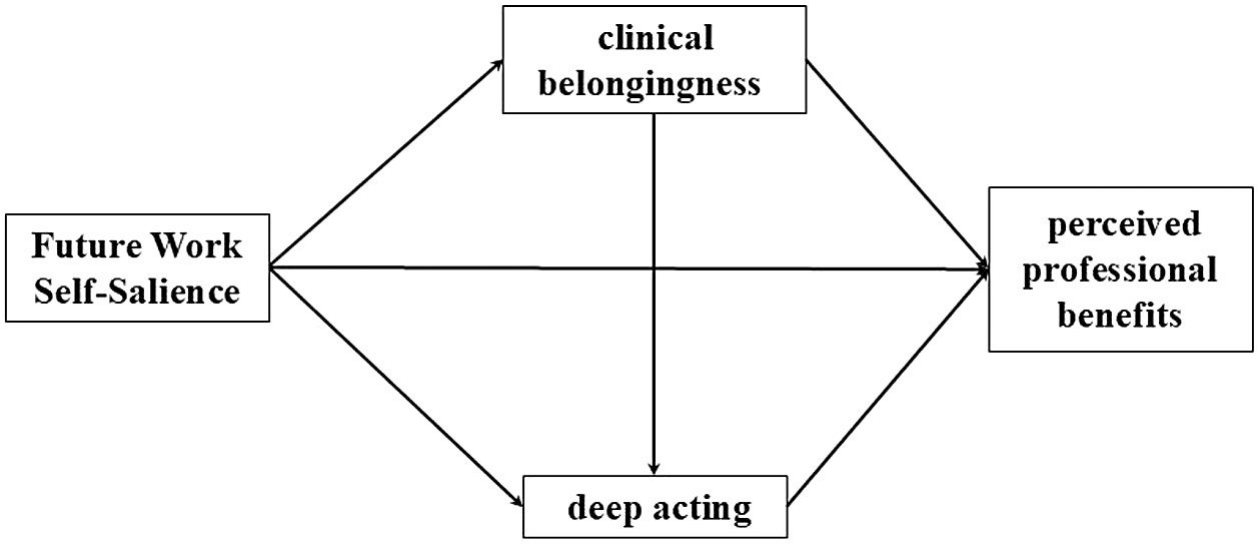
Hypothesized chain mediation model in which future work self-salience is associated with perceived professional benefits through clinical belongingness and deep acting.

**Figure 2:**
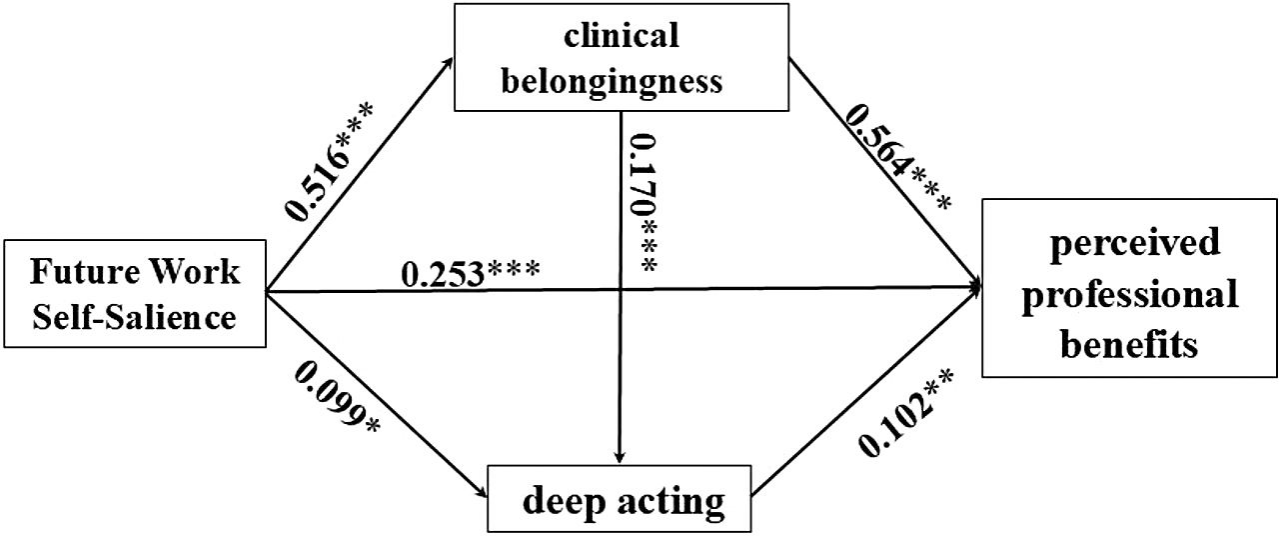
Standardized path coefficients for the chain mediation model. Six demographic covariates (residence, preference for nursing, academic ranking, Average monthly household income, employment prospect, and highest education level) were controlled but not shown for simplicity. *p < 0.05, **p < 0.01, ***p < 0.001.

All 95% confidence intervals excluded zero, confirming the significance of each indirect pathway. Figures 1,2 present the hypothesized and tested models.

## 4 Discussion

### 4.1 Principal findings

This cross-sectional study examined the associations among future work self-salience, clinical belongingness, deep acting, and perceived professional benefits among nursing interns in Guangxi, China. The results showed that future work self-salience was positively associated with perceived professional benefits. In the hypothesized SEM model, clinical belongingness and deep acting were involved in significant indirect associations, including a sequential pathway from clinical belongingness to deep acting. These findings should be interpreted as correlational and theory-driven rather than causal.

### 4.2 Future work self-salience as a motivational resource for perceived professional benefits

The positive association between future work self-salience and perceived professional benefits aligns with broader research showing that career-related clarity and future-oriented self-representations support goal commitment, persistence, and adaptive coping (49). Interns who can vividly envision their future professional selves may be more likely to interpret routine clinical tasks—documentation, basic care, repeated practice of procedures—as meaningful investments rather than burdensome requirements (50). This meaning-making process can enhance satisfaction and strengthen the perceived value of nursing work, thereby increasing perceived professional benefits. future work self-salience may also reduce uncertainty and role ambiguity by providing a cognitive map for “where I am going” and “why this effort matters,” which is particularly important during internships characterized by unfamiliar environments, shifting expectations, and frequent performance evaluation (51, 52). In addition, clearer future selves may foster proactive behaviors that improve competence development and social recognition—both central components of perceived professional benefits (53). From this perspective, future work self-salience may function as an upstream psychological resource that shapes how interns appraise stressors and rewards during the transition into practice.

### 4.3 The mediating role of clinical belongingness

Clinical belongingness emerged as a strong mediator, underscoring the centrality of the clinical learning environment in shaping interns' professional appraisals (54). Belongingness is widely recognized as a fundamental psychological need, and its fulfillment in educational and workplace settings has been linked to engagement, self-efficacy, well-being, and persistence. In clinical internships, belongingness is cultivated through respectful interactions, inclusive team practices, supportive supervision, and opportunities for legitimate participation in patient care. When interns feel accepted and valued, they are more likely to experience psychological safety, which encourages learning-oriented behaviors, reduces fear of negative evaluation, and supports adaptive responses to mistakes and feedback (11, 55). These experiences can translate into higher perceived professional benefits by strengthening professional meaning, increasing confidence in professional fit, and enhancing perceptions of growth and social recognition (22).

The present findings also suggest a plausible “agency-to-context” pathway: interns with clearer future work selves may be more motivated to build professional relationships, integrate into clinical teams, and persist in seeking support despite initial barriers. Future work self-salience may therefore facilitate belongingness by promoting intentional engagement with the clinical environment. This interpretation aligns with research indicating that proactive career cognition and goal clarity are associated with greater help-seeking, relationship-building, and adaptive social behaviors in training contexts. In contrast, interns who lack a clear future work self may experience clinical stressors as disconnected from personal goals, potentially reducing motivation to invest in team integration and increasing vulnerability to isolation (49, 56, 57).

### 4.4 The mediating role of deep acting and its professional significance

The present study focused specifically on the deep acting dimension of emotional labor rather than treating it as a unitary construct. This approach reflects the substantial heterogeneity among emotional labor strategies: surface acting is negatively associated with job satisfaction and linked to emotional exhaustion and burnout, whereas deep acting is positively associated with job satisfaction and has been related to nursing professionalism and higher-quality interpersonal interactions (58–60). Perceived professional benefits, as a positive occupational outcome reflecting professional growth and identity, is theoretically more aligned with the “genuine emotional regulation” pathway represented by deep acting than with the “emotional faking” pathway of surface acting. From the perspective of conservation of resources theory (61), deep acting may be associated with greater congruence between emotional expression and inner experience through cognitive reappraisal, which may help reduce resource depletion and support the accumulation of positive psychological resources (59, 62).

Deep acting mediated the link between future work self-salience and perceived professional benefits, highlighting the importance of emotion regulation in early professional development. Nursing interns routinely encounter emotionally charged situations, including patient suffering, time pressure, and complex interpersonal interactions (63–65). Deep acting—attempting to genuinely feel the emotions required by the role—has been associated with more authentic caregiving, improved interpersonal outcomes, and better well-being compared with surface acting (42, 66, 67). Interns who engage in deep acting may experience greater congruence between internal feelings and professional expressions, reducing emotional dissonance and facilitating a sense of competence in relational care (32). These experiences likely contribute to perceived professional benefits by reinforcing the meaningfulness of nursing work and strengthening professional identity. Although deep acting is often considered adaptive, it may still involve regulatory effort; therefore, the positive association observed here should not be interpreted as implying that greater emotional regulation demands are universally beneficial.

Future work self-salience may support deep acting in several ways. A clear future professional self can increase purpose-driven motivation, making it easier to invest effort in emotionally demanding interactions that are interpreted as integral to becoming the desired nurse (49). It may also enhance self-regulatory capacity when emotions conflict with professional expectations (56), and facilitate adaptive coping and reflective practices (42). Together, these processes suggest that future-oriented clarity supports not only clinical learning behaviors but also how interns regulate emotions during practice. Nevertheless, given the cross-sectional nature of this study, the causal ordering between these variables remains speculative and should be examined in future longitudinal or experimental designs.

### 4.5 The sequential pathway from belongingness to deep acting

Beyond independent mediation, the supported chain pathway provides a more integrated account of how future-oriented cognition translates into professional gains. One interpretation is that clinical belongingness creates relational and emotional resources—supportive supervision, peer connection, psychological safety, and a sense of legitimacy—that increase interns' capacity to engage in deep acting (68). Deep acting often requires cognitive effort, emotional awareness, and motivation to align inner feelings with professional values; these capacities may be strengthened when interns feel supported and included rather than threatened or marginalized (69). Belongingness may also reduce stress and defensive coping, making it more likely that interns approach patient interactions with openness and empathy rather than emotional withdrawal (55).

This sequential process is consistent with resource-based models of adaptation (70), which propose that resources accumulate in gain spirals: initial resources are associated with contextual resources, which then enable more demanding adaptive strategies, ultimately showing positive associations with outcomes. The present findings therefore extend prior research by empirically connecting career-related clarity, clinical social integration, and emotional labor strategy within one explanatory framework in a nursing internship population.

### 4.6 Practical implications for nursing education and clinical management

These findings suggest several potential directions for nursing educators, clinical preceptors, and administrators.

First, career-vision activities, goal-setting exercises, and individualized career counseling may help interns clarify their professional goals and connect daily learning with future development (71, 72). However, given the cross-sectional design of this study, the present findings cannot demonstrate the actual effectiveness of these strategies, which should be examined in future longitudinal or intervention studies.

Second, clinical belongingness likely constitutes a key dimension of the training environment. Clinical units can foster belongingness by clarifying role expectations, encouraging respectful communication, and giving interns appropriate opportunities to engage in patient care (73–75). Educators and unit leaders may also consider strengthening inclusive supervision and educational support from clinical staff.

Third, emotion regulation and emotional labor may also deserve attention in internship curricula. Training in emotional awareness, reflective practice, empathic communication, and adaptive regulation strategies may help interns better manage emotional demands in clinical settings (33, 76, 77). Such efforts may be more meaningful when combined with a supportive clinical environment.

Finally, these findings suggest that future programs may benefit from an integrated focus on career development, team inclusion, and emotional adaptation. However, whether such approaches improve interns' perceived professional benefits should be confirmed in future research.

### 4.7 Limitations and future directions

Several limitations should be acknowledged. First, the cross-sectional design limits causal inference; longitudinal studies are needed to examine how future work self-salience, belongingness, deep acting, and perceived professional benefits co-evolve across internship stages and whether changes in future work self-salience are associated with subsequent changes in mediators and outcomes. Second, the study relied on self-report measures, which may be related to social desirability and shared method variance. Future research could incorporate multi-source assessments, such as preceptor ratings, peer reports, behavioral indicators, or objective measures related to clinical engagement and emotional demands. Third, the sample was drawn from a specific regional context and used convenience sampling, which may limit generalizability. Replication across diverse institutions, cultural settings, and internship systems would strengthen external validity. These institutions were selected based on their established internship collaboration units, their capacity to accommodate nursing interns in clinical training, and the administrative feasibility of conducting the survey during the study period. Consequently, institutional selection was guided by accessibility and practical feasibility rather than by random or stratified sampling. Fourth, other theoretically relevant variables, such as professional identity, self-efficacy, psychological capital, and perceived clinical learning environment, were not included and may partly account for the observed associations.

Future work could also explore boundary conditions and additional processes. Potential moderators include internship department, workload intensity, shift patterns, preceptor leadership style, perceived organizational support, and individual traits such as resilience, emotion regulation ability, and professional calling. Additional mediators may include self-efficacy, professional identity, psychological capital, coping strategies, and learning engagement. Moreover, intervention and quasi-experimental designs would be valuable for testing whether enhancing future work self-salience or improving belongingness climates produces downstream gains in deep acting and perceived professional benefits.

## 5 Conclusion

Future work self-salience was positively associated with perceived professional benefits among nursing interns in Guangxi, China. In the hypothesized cross-sectional model, clinical belongingness and deep acting were involved in significant indirect associations, including a sequential pathway from clinical belongingness to deep acting. These findings suggest that future career clarity, inclusive clinical environments, and adaptive emotional labor may be relevant factors for nursing interns' positive professional perceptions. Longitudinal and intervention studies are needed to determine whether modifying these factors can improve professional benefits and support nursing workforce development.

## Data Availability

The raw data supporting the conclusions of this article will be made available by the authors, without undue reservation.
